# Gas Generation Characteristics and Mechanisms of Medium-Rank Bituminous Coal Under Rotational Shear

**DOI:** 10.3390/nano16201287

**Published:** 2026-10-10

**Authors:** Nannan Cheng, Xian Wang, Wenhui Yan, Mengyan Shi, Jienan Pan, Quanlin Hou

**Affiliations:** 1 School of Resources and Environment, Henan Polytechnic University, Jiaozuo 454003, China; chengnannan@hpu.edu.cn (N.C.); jn_pan@hpu.edu.cn (J.P.); 2 Henan Key Laboratory of Coal Measure Unconventional Resources Accumulation and Exploitation, Jiaozuo 454003, China; 3 College of Earth and Planetary Sciences, University of Chinese Academy of Sciences, Beijing 100049, China; quhou@ucas.ac.cn

**Keywords:** coal chemical structure, stress polycondensation, tectonic stress chemistry, gas generation mechanism

## Abstract

Clarifying whether tectonic stress directly induces coal macromolecular structural evolution and gas generation plays a key role in elucidating deep coal gas sources and coal-gas outburst mechanisms. Existing studies pay insufficient attention to medium-rank bituminous coal, which is abundant in aliphatic side chains and active functional groups, and its stress-responsive gas generation mechanism remains unclear. Rotational shear experiments were conducted on coal from Pingdingshan No.10 Mine under 12.5 MPa axial pressure at 25 °C and 80 °C with three shear rates. Notably, no replicate samples were set for each experimental condition. Gas generation characteristics and structural evolution were characterized via FTIR, ^13^C NMR and GC–MS analyses. Results show that 25 °C shearing only causes physical fragmentation, with CO_2_ as the sole product. Under 80 °C thermo-mechanical coupling, low shear rate preferentially cleaves aliphatic side chains, yielding the highest CH_4_ and promoting aromatic polycondensation. High shear rate fragments aromatic skeletons, suppressing hydrocarbon generation and dominating CO_2_ production. This study verifies the stress–temperature coupling chemical effect of medium-rank bituminous coal and reveals the shear-rate-regulated differential gas generation mechanism, providing direct experimental evidence for tectonic stress-induced coalification and gas generation.

## 1. Introduction

Coal represents a distinct type of organic sedimentary rock whose architecture is built upon stacked aromatic sheets. Within this framework, polycyclic aromatic hydrocarbon units are linked together by aliphatic side chains, various functional groups, and aliphatic carbon bridges, giving rise to a robust macromolecular network [1,2]. Owing to its inherently low mechanical competence, coal responds readily to applied stress and strain, such that both its physical fabric and chemical constitution can be modified when subjected to tectonic loading. Gaining insight into these structural alterations is essential for deciphering the mechanisms governing coalification and coalbed methane generation. Conventionally, temperature and geological time have been regarded as the principal factors determining the rank of coalification [3,4,5,6]. The role of tectonic stress, by contrast, has largely been considered limited to modifying the physical attributes of coal, particularly its pore architecture and fracture system [7,8,9,10], while its capacity to drive genuine chemical restructuring within the coal matrix continues to be a subject of controversy [11,12]. Nevertheless, coal and gas outbursts are persistently observed in seams cut by faults and shear zones, and the quantity of gas liberated during such events frequently surpasses the maximum adsorption capacity of the coal involved [13,14]. This discrepancy poses a pivotal question: is tectonic stress capable of rupturing chemical bonds in coal, initiating chemical reactions, and consequently inducing hydrocarbon generation or gas emission? This issue has drawn growing scholarly interest in recent years [15,16,17,18].

Fourier transform infrared spectroscopy (FTIR) and ^13^C NMR analyses comparing tectonically deformed coals with their undeformed counterparts of the same coal rank have revealed that deformed coals contain lower proportions of aliphatic structures and higher proportions of aromatic structures [19,20,21,22]. On this basis, two mechanisms have been proposed to explain how tectonic stress influences the coalification process: stress degradation and stress condensation [23,24]. On one hand, tectonic stress promotes the dissociation of aliphatic chains or functional groups into free radicals or small molecules. On the other hand, stress can facilitate the condensation and expansion of aromatic sheets. Nevertheless, the detailed mechanisms governing chemical bond cleavage and aromatic structure growth in coal under tectonic stress remain poorly understood.

To further explore the chemical structural changes and gas generation mechanisms of coal under tectonic stress, our research group has conducted a series of deformation experiments and computational simulations. The results demonstrated that gas generation occurred under stress at temperatures below 200 °C, with different coal ranks producing different gases under varying experimental conditions. For instance, medium-rank coking coal generated CH_4_ at 70–100 °C [25]; high-rank anthracite produced CO_2_ and CH_4_ at 200 °C under stress parallel to the bedding plane, while the same high-rank anthracite generated CO at 100–200 °C under low strain rates [26]. FTIR and ^13^C NMR analyses of these deformed coal samples, conducted by Xu et al. [27] and Han et al. [28] revealed that the deformed samples exhibited significantly reduced methyl content, a progressive decline in aliphatic structures and oxygen-containing functional groups, a gradual increase in aromatic structures, and the development of abundant structural defects. Computational simulations of anthracite deformation under stress also showed that the mechanical energy applied during deformation experiments could be converted into strain energy, promoting the distortion and disruption of coal molecular units, and that the structural defects present in ductile-deformed coals could lower the energy required for chemical bond cleavage [29,30]. Further calculations indicated that tectonic stress could directly break or distort the chemical bonds of macromolecular structures [31,32] and even entire fused aromatic rings [33], thereby promoting the generation of gases such as H_2_, CO, CO_2_ and CH_4_ [34,35], with shearing stress being far more efficient at bond cleavage than normal stress [36,37]. Despite the substantial progress that these investigations have yielded in elucidating the mechanochemical behavior of coal subjected to tectonic stress, robust and direct empirical evidence demonstrating the capacity of tectonic stress to rupture chemical bonds and thereby facilitate gas generation remains scarce.

Coal rank determines the distribution of functional groups within its macromolecules, which in turn governs the gas generation potential during tectonic deformation. As the degree of coal metamorphism increases, the proportion of oxygen-containing functional groups gradually decreases while the proportion of aromatic structures increases. Experimental and theoretical studies have shown [38,39] that medium-rank coals retain abundant aliphatic side chains and a moderate aromatic nucleus structure, making them more susceptible to stretching and shearing fracture of aliphatic chains under tectonic stress, thereby generating small molecule hydrocarbon gases. Although preliminary computational simulations have been conducted on the gas generation mechanism of medium-rank coal deformation under shearing [34,35], systematic investigation from the perspective of deformation experiments is still needed to address the feasibility and characteristics of gas generation in medium-rank coal under shear stress, the evolution of macromolecular structures in deformed coal, and the mechanochemical reaction mechanisms induced by tectonic stress.

To address these knowledge gaps, the current paper is devoted to medium-rank bituminous coal collected in the No. 10 Mine of the Pingdingshan Coalfield. A set of rotational shear deformation experiments was conducted under a combination of controlled conditions, including two temperatures (25 °C and 80 °C) and three shear rates (0.14, 0.69, and 3.44 °/min). Through a systematic study of the mechanisms underlying the control of gas generation behavior and macromolecular structural evolution by stress–temperature coupling in medium-rank bituminous coal, we combined GC–MS, FTIR and ^13^C NMR spectroscopic analysis. The results of this research are added to the body of knowledge on coalification theory and provide a theoretical framework to explain the origin of abnormal gas occurrences in coal mines and to prevent coal and gas outbursts.

## 2. Deformation Experiments and Analytical Procedures

### 2.1. Sample Collection and Preparation

**Figure 1:**
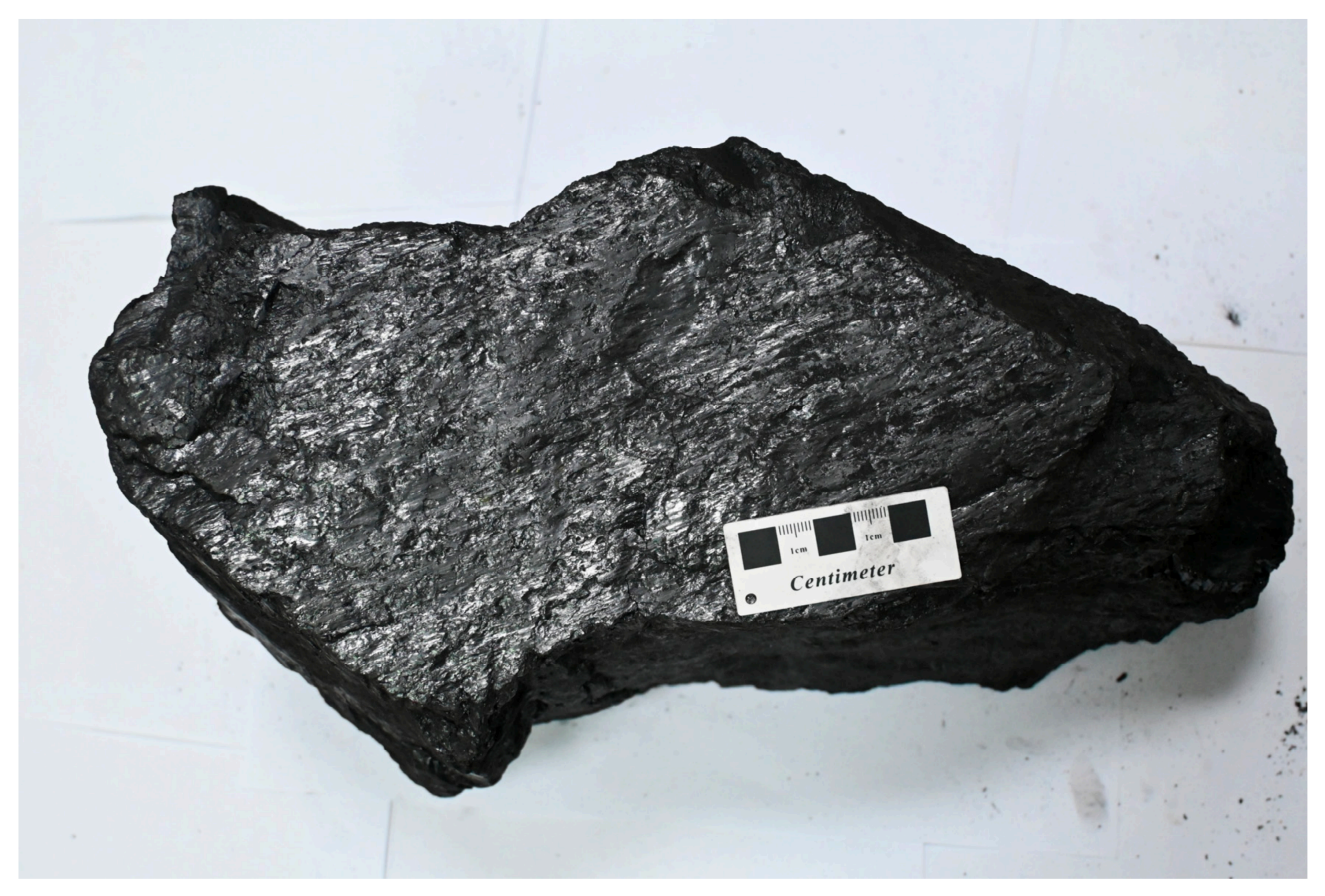
Hand specimen photo of the raw coal sample.

The coal samples were collected from the #4_2_ coal seam at the No. 10 Mine, Pingdingshan Coalfield, Henan Province (Figure 1). The samples were subjected to proximate and ultimate analyses, and the results are shown in Table 1. Based on the standard coal-rank classification system, the coal is medium-rank bituminous coal. Following sampling, the coal samples were kept under ambient atmospheric conditions for four months to ensure full desorption of the natural gas.

### 2.2. Experimental Apparatus and Procedures

**Figure 2:**
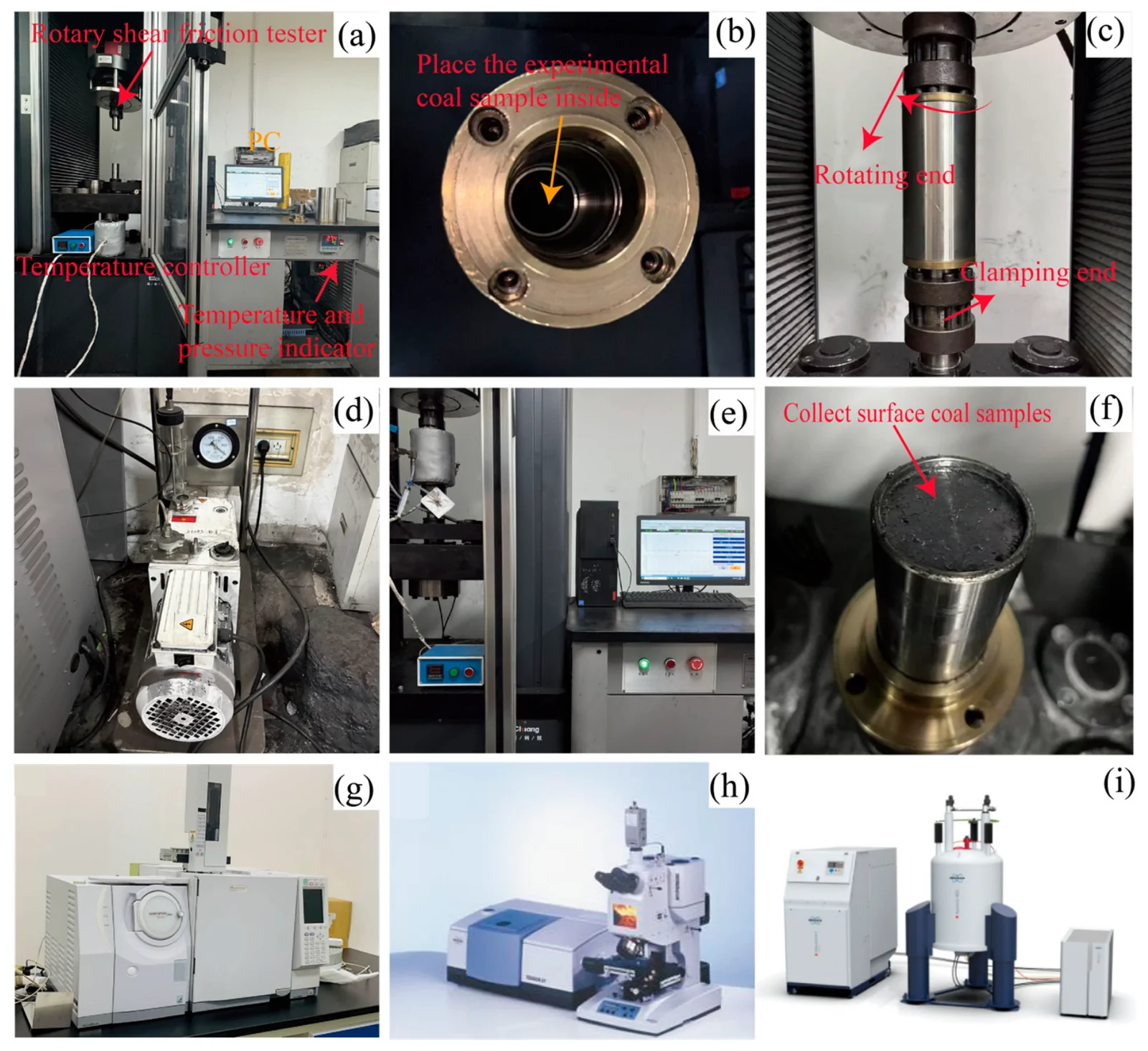
Rotational shear deformation apparatus and operating procedure. (**a**) Panoramic view of the experimental apparatus; (**b**) Photograph of the sample placed in the sealed tank; (**c**) Schematic diagram of the sealed tank during operation; (**d**) Photograph of the vacuum pump; (**e**) Panoramic view of the fully installed apparatus; (**f**) Photograph of the sample after experiment; (**g**) Photograph of the GC–MS instrument; (**h**) Photograph of the FTIR instrument; (**i**) Photograph of the ^13^C NMR instrument.

Experiments of rotational shear deformation were conducted at Henan Polytechnic University using a coal-rock rotational shear friction testing machine (Figure 2a). The sample groove in a hermetically sealed canister contained approximately 13 g of coal specimen (Figure 2b). The canister, along with the lower plunger and upper piston was fastened onto the test platform (Figure 2c). Gas sampling bags, pressure transducers and vacuum pump were also provided to the experimental set up. Before starting the test, the canister was pumped out through the vacuum pump (Figure 2d) and then the test parameters were set (Figure 2e). During the shearing process, the changes in pressure in the system were measured in real time. The pressure transducer employed had a full measurement range of 0–0.2 MPa and an accuracy grade of 0.5, with an absolute measurement uncertainty of ±0.0005 MPa. After every experiment, gas samples and the pulverized coal obtained after removing the upper layer (Figure 2f) were retrieved to be characterized by further GC–MS (Figure 2g), FTIR (Figure 2h), and ^13^C NMR (Figure 2i) characterization, thus making sure that all materials exposed to analysis had undergone active shear deformation.

To eliminate interference from pre-adsorbed gases, raw coal samples were ambiently desorbed for four months to ensure complete gas release. The coal was ground to 40 mesh to unify grain size. Samples were dried in a vacuum oven at 60 °C for 12 h prior to testing.

Experimental runs were separated into a sub-high temperature group (80 °C, Samples A1–A3) and room-temperature group (25 °C, Samples A4–A6), where raw coal was labeled as A0. Three shear rates were chosen based on natural fault strain rate ranges (10^−8^–10^−5^/s^−1^), coal rheological testing strain rates (2.5–20 × 10^−6^/s^−1^), and apparatus capabilities (1–40 × 10^−6^ m/s^−1^): low (0.14 °/min), medium (0.69 °/min), and high (3.44 °/min). The rotational shear was combined with a constant axial pressure of 12.5 MPa to replicate the coupled normal and shear stress state of natural deformation zones. Time-dependent deformation was considered by setting testing duration to 48 h. Uniaxial compression desorption controls (Samples B1 and B2) were performed in the absence of shear to isolate gas release that is purely pressure-induced. Table 2 summarizes the experimental matrix.

### 2.3. GC–MS

GC–MS analysis was conducted at Hangzhou Xuyan Technology Studio, with an Agilent 7820A-5977E GC–MS instrument, with procedures adjusted based on the earlier established protocols [40,41]. The separation by chromatography was done using an HP-5 fused-silica capillary column (30 m × 0.25 mm × 0.25 μm). The temperature of the injector was kept constant at 300 °C and hold time was 10 min. Nitrogen (99.99%) was used as the carrier gas and was supplied at a steady flow rate of 3 mL/min.

Since the experimental system is run under relatively small pressure variations in both ambient and sub-high temperature conditions, and since the gaseous products involved are dominated by light hydrocarbons and inorganic gases, it is reasonable to approximate the gas phase as an ideal gas. Assuming negligible intermolecular forces and molecular volume, the cumulative gas yield was determined with the help of the ideal gas law:

(1) PV = nRT

where *P* is the change in pressure (Pa); *V* is the net volume remaining in the sealed canister after subtracting the metallic grooves and is approximately 16.62 cm^3^; R is the universal gas constant (8.314 Pa m^3^/(mol. K)); and *T* is the absolute temperature of the experiment (*T* = 273 + *t*, where *t* is the temperature in Celsius).

The total gas volume was calculated using the following equation

(2) $$V_{\mathrm{total}}={\;n}_{\mathrm{total}}\times \;V_{m,std}$$

where *V*_m,std_ denotes the molar volume of gas under standard temperature and pressure (22,400 mL/mol).

The volume of each individual gas component was calculated using the following equation:

(3) $$V_{\mathrm{single}\;\mathrm{gas}\;}=\;V_{\mathrm{total}}\times \;\frac{\mathrm{Fraction}\;\mathrm{of}\;\mathrm{individual}\;\mathrm{gas}}{\mathrm{Total}\;\mathrm{fraction}}$$

### 2.4. FTIR

**Figure 3:**
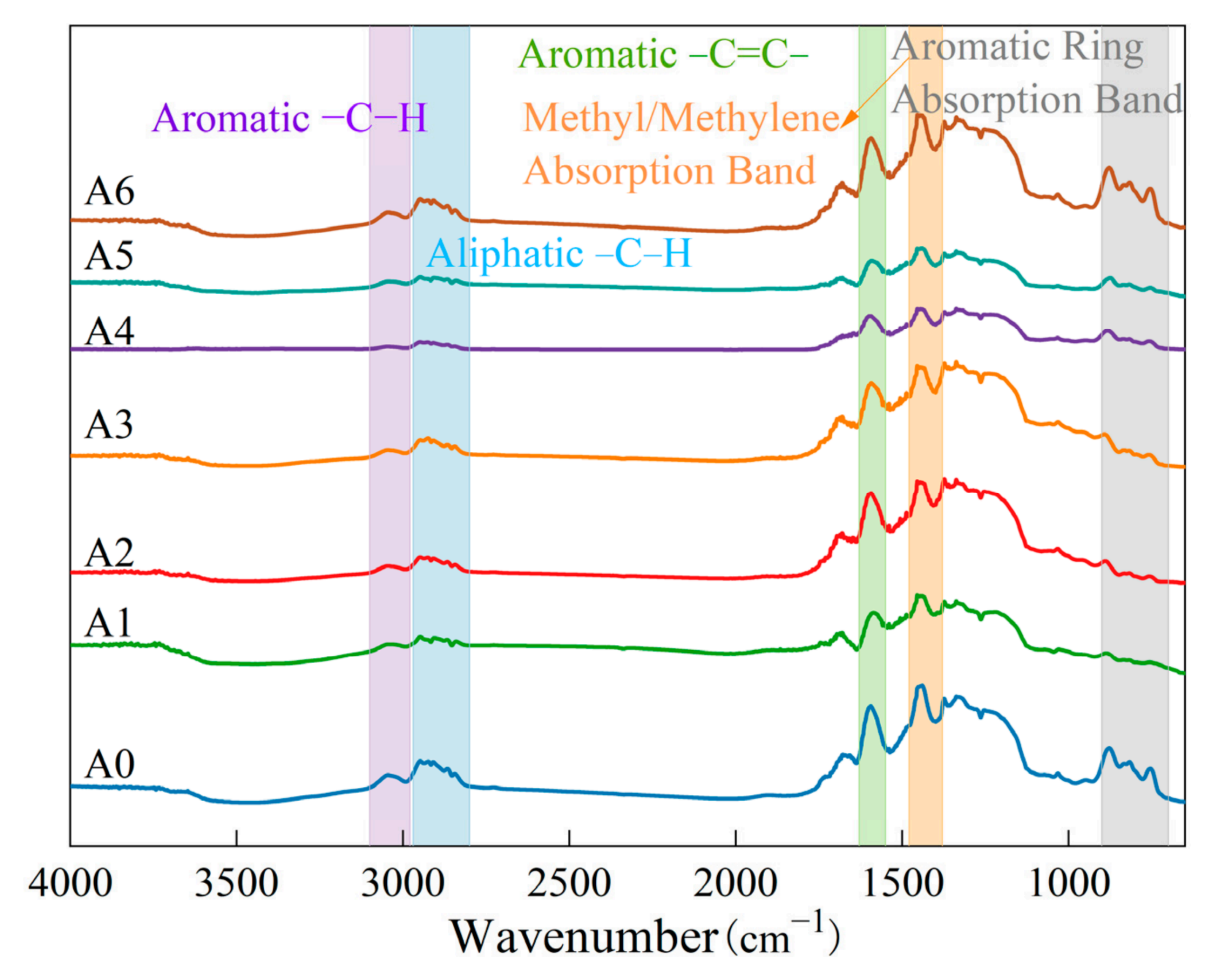
FTIR spectra of the raw coal and the experimentally deformed samples.

FTIR analysis was performed using a Nicolet 6700 spectrometer. Coal samples were ground below 200 mesh, demineralized with acid, vacuum-dried, and mixed with KBr at a 1:199 mass ratio before being pressed into translucent pellets. Spectra were recorded across 400–4000 cm^−1^. Peak fitting was conducted using Origin software with an iterative Gaussian–Lorentzian mixed function across five spectral regions: 700–900 cm^−1^, 1380–1480 cm^−1^, 1550–1630 cm^−1^, 2800–2970 cm^−1^ and 2980–3070 cm^−1^ (Figure 3). Within each region, the number and assignment of peaks were determined based on the corresponding types of functional groups.

### 2.5. ^13^C NMR

**Figure 4:**
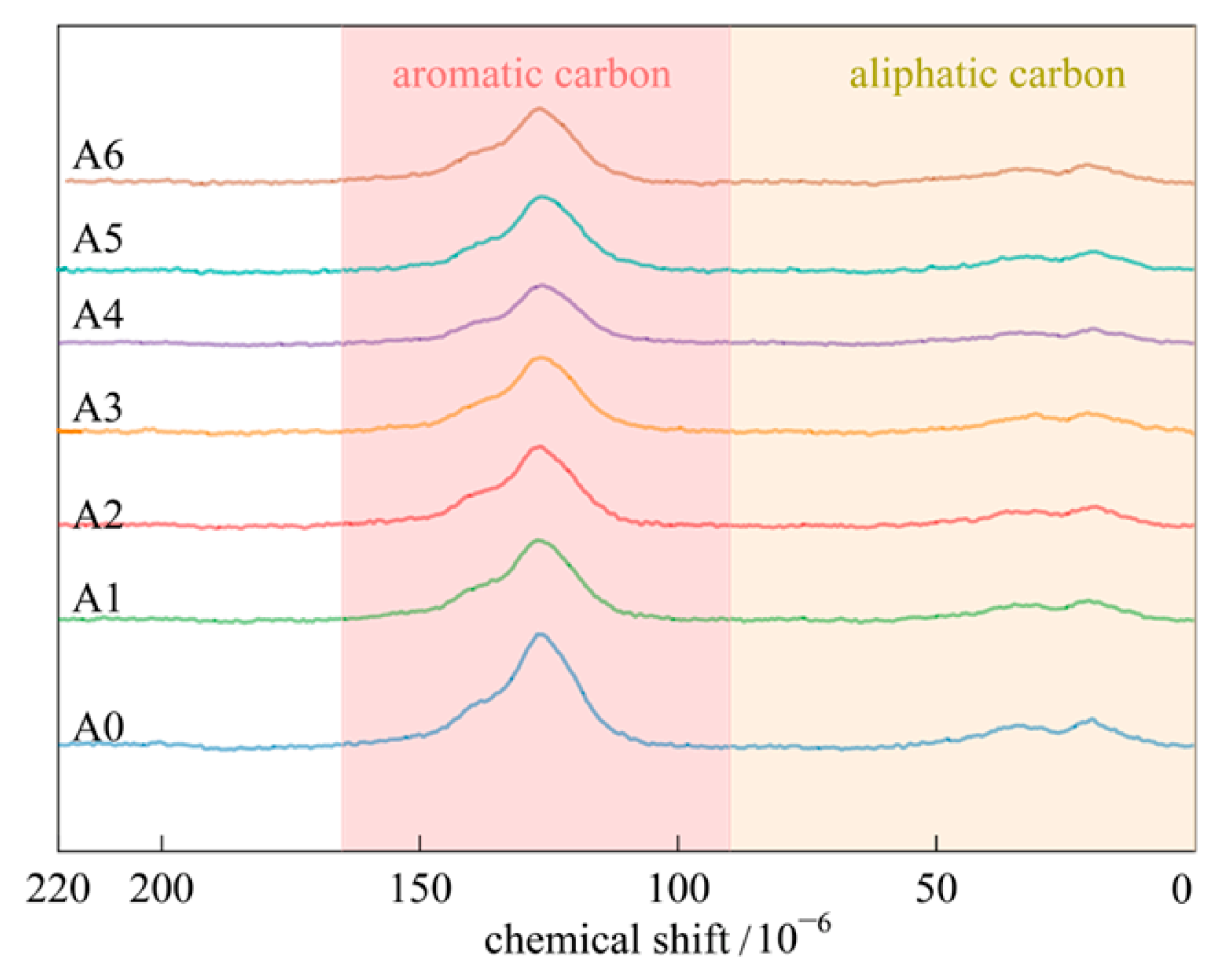
Summary of ^13^C NMR spectra for the raw coal and deformed samples.

Solid-state ^13^C NMR spectra were acquired on an Agilent 600M spectrometer (Agilent Technologies, Inc., Santa Clara, CA, USA) using a 4 mm zirconia rotor magic-angle spinning (MAS) probe. Operating parameters included a spinning speed of 8 kHz, a recycle delay of 5 s, 4000 scans, a spectral width of 30 kHz, and a contact time of 3 ms, referenced to tetramethylsilane (TMS, 0 ppm). Peak deconvolution was performed in Origin across aliphatic (0–90 ppm) and aromatic (90–165 ppm) regions (Figure 4). Within each region, the number and assignment of peaks were determined based on the corresponding types of functional groups.

## 3. Results and Analysis

### 3.1. Gas Generation Characteristics in Rotating Shear Deformation Experiments

**Figure 5:**
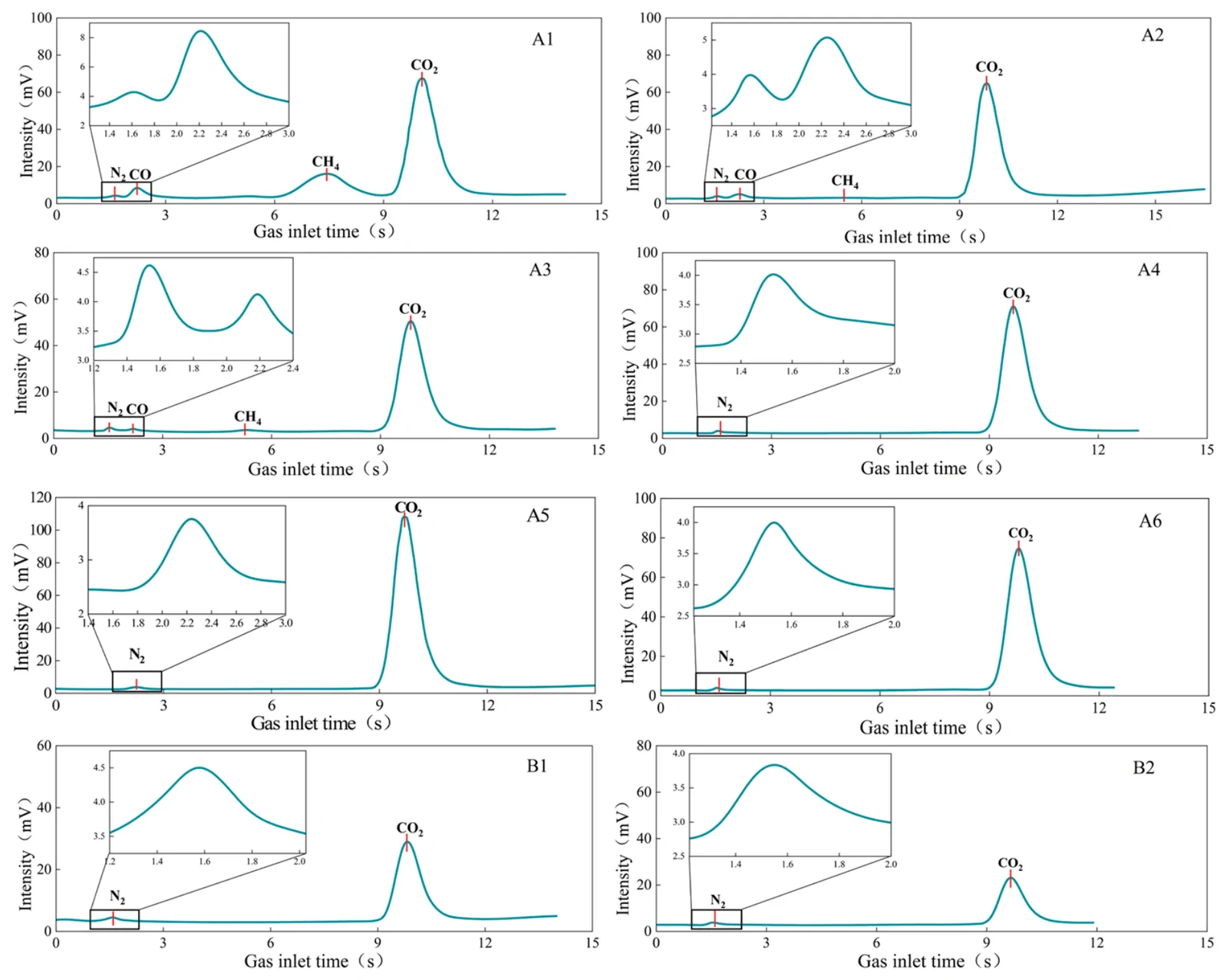
GC–MS chromatograms.

**Figure 6:**
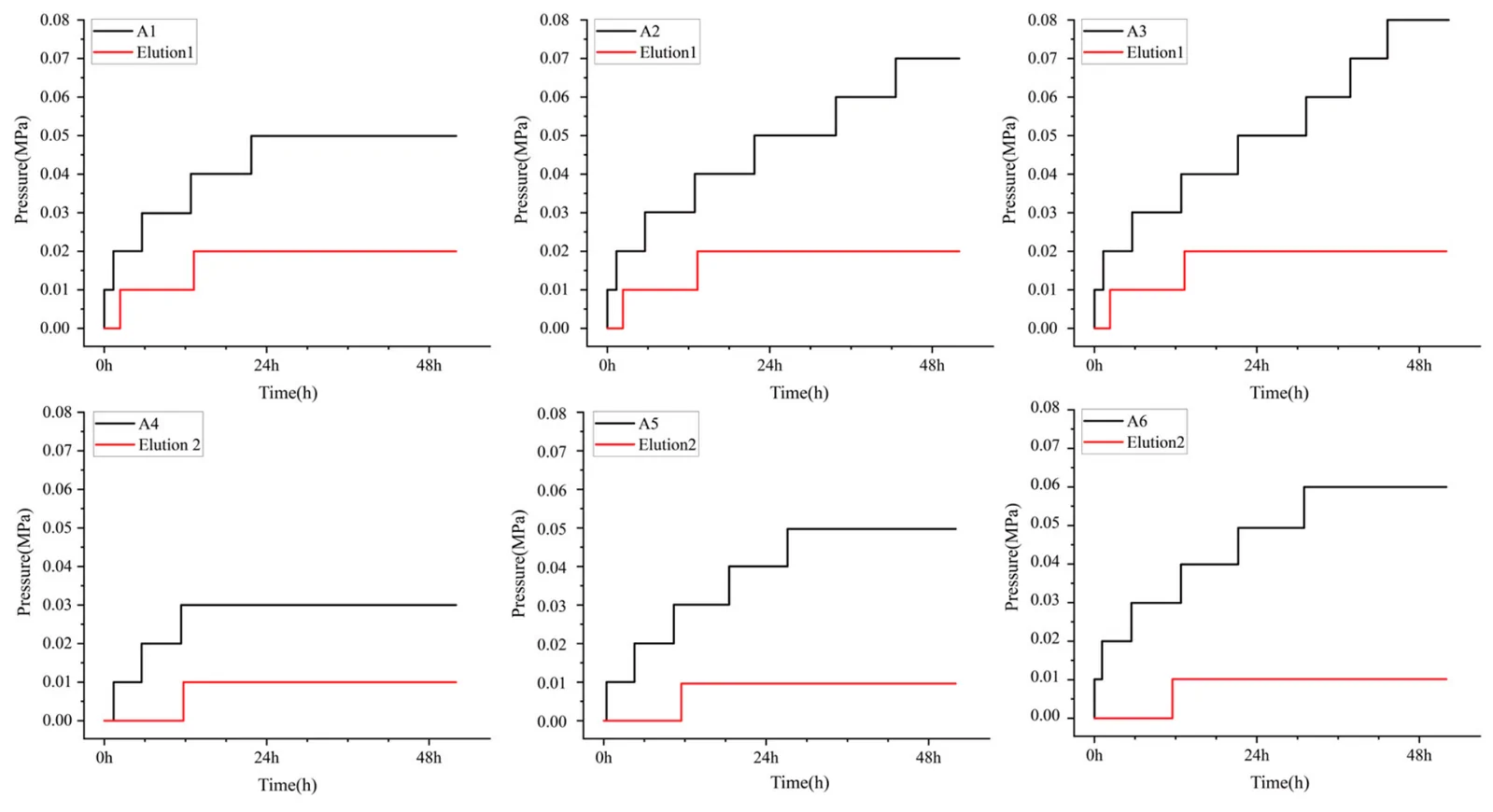
Pressure variation curves during the rotational shear experiments.

GC–MS analyses (Figure 5) and real-time pressure sensor recordings (Figure 6) recorded baseline pressure variations of 0.02 MPa and 0.01 MPa in the uniaxial compression desorption controls (B1 and B2, respectively). Subtracting these baseline desorption values yielded net pressure changes for the shear deformation experiments.

Sample A1 under the thermal condition of 80 °C recorded a net pressure increase of 0.03 MPa with the gaseous products being CO, CH_4_ and CO_2_ in a volumetric ratio of 1:6.7:20. In sample A2, the pressure rose by 0.05 MPa and the gases evolved had moderate amounts of CO and CO_2_ with a small amount of CH_4_ (volumetric ratio of 1:7.5:310.5). Sample A3 had the largest increment of pressure of 0.06 MPa among the 80 °C group, producing significant quantities of CO and CO_2_ but only traces of CH_4_ (volumetric ratio of 1:1.3:2094). In comparison, the experiments at 25 °C resulted in the formation of CO_2_ as the only gaseous species, and the net increases in pressure were 0.02 MPa (A4), 0.04 MPa (A5), and 0.05 MPa (A6), respectively.

According to the pressure dynamics and Equations (1)–(3), the quantitative gas yields were calculated and are summarized in Table 3. At 80 °C, sample A1 produced a cumulative volume of gas of 4.15 mL comprising 0.15 mL CO, 1.00 mL CH_4_ and 3.00 mL CO_2_. In the case of sample A2, the overall gas production increased to 6.92 mL, with 0.02 mL CO, 0.16 mL CH_4_ and 6.74 mL CO_2_. The largest total gas volume of 8.31 mL was obtained in sample A3, where CO_2_ dominated by far with 8.30 mL, followed by marginal contributions of CO (0.004 mL) and CH_4_(0.0052 mL). The desorption control sample B1 emitted 2.77 mL of CO_2_. As shear rate increased between A1 and A3, the yields of both CO and CH_4_ declined gradually, e.g., CO declining between 0.15 mL and 0.004 mL and CH_4_ declining drastically between 1.00 mL and 0.0052 mL. Conversely, the amount of CO_2_ showed a steady upward trend, increasing to 8.30 mL as compared to 3.00 mL.

At 25 °C, CO_2_ remained the only evolved gas species. Generated CO_2_ volumes increased progressively from 3.28 mL in A4 to 6.56 mL in A5 and 8.20 mL in A6, compared to 1.64 mL produced by the room-temperature desorption control B2. These data demonstrate a direct correlation between shear rate and total gas evolution under ambient temperature conditions.

In terms of gas composition patterns, the 80 °C experimental group developed mixed gas products with relative proportions varying significantly with shear rate. CH_4_ and CO yields were found to be maximum at A1 (low-rate shear) of 1.00 mL and 0.15 mL, respectively. With high-rate shear (A3), CH_4_ and CO decreased to trace levels, whereas CO_2_ volume increased from 3.00 mL to 8.30 mL. These results suggest that low shear rates in combination with sub-high temperatures are the best conditions under which hydrocarbon gases can be generated and released out of the coal structure. In both thermal regimes, an increase in shear rates was always associated with an increase in the total overall volume of gas released.

### 3.2. Chemical Structure Evolution Under Sub-High Temperature Conditions

**Figure 7:**
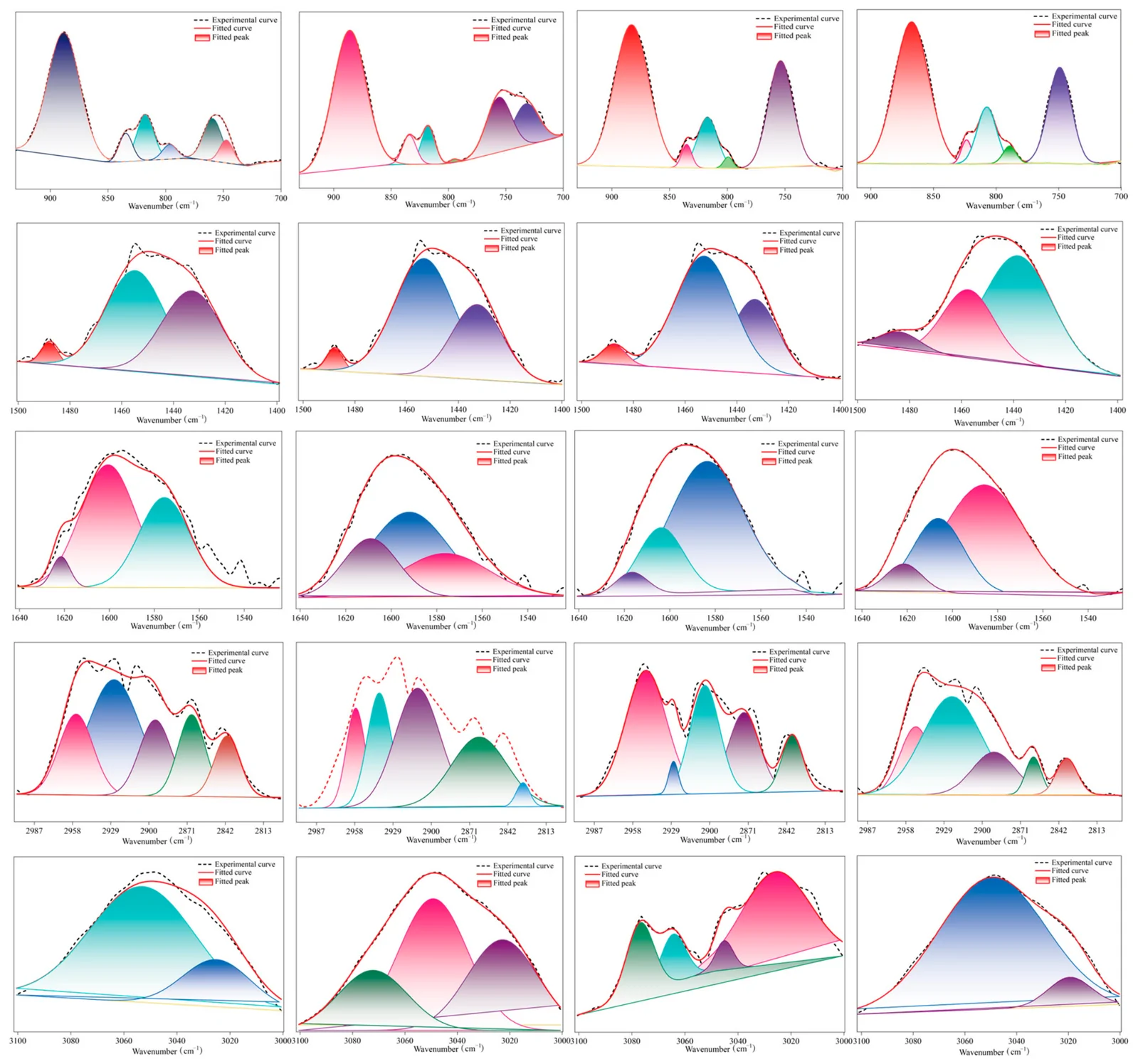
Percentages and ratios of different FTIR absorption bands for the raw coal and sub-high temperature samples (from left to right, columns 1 to 4 are A1, A2, A3, and A0 in sequence).

**Figure 8:**
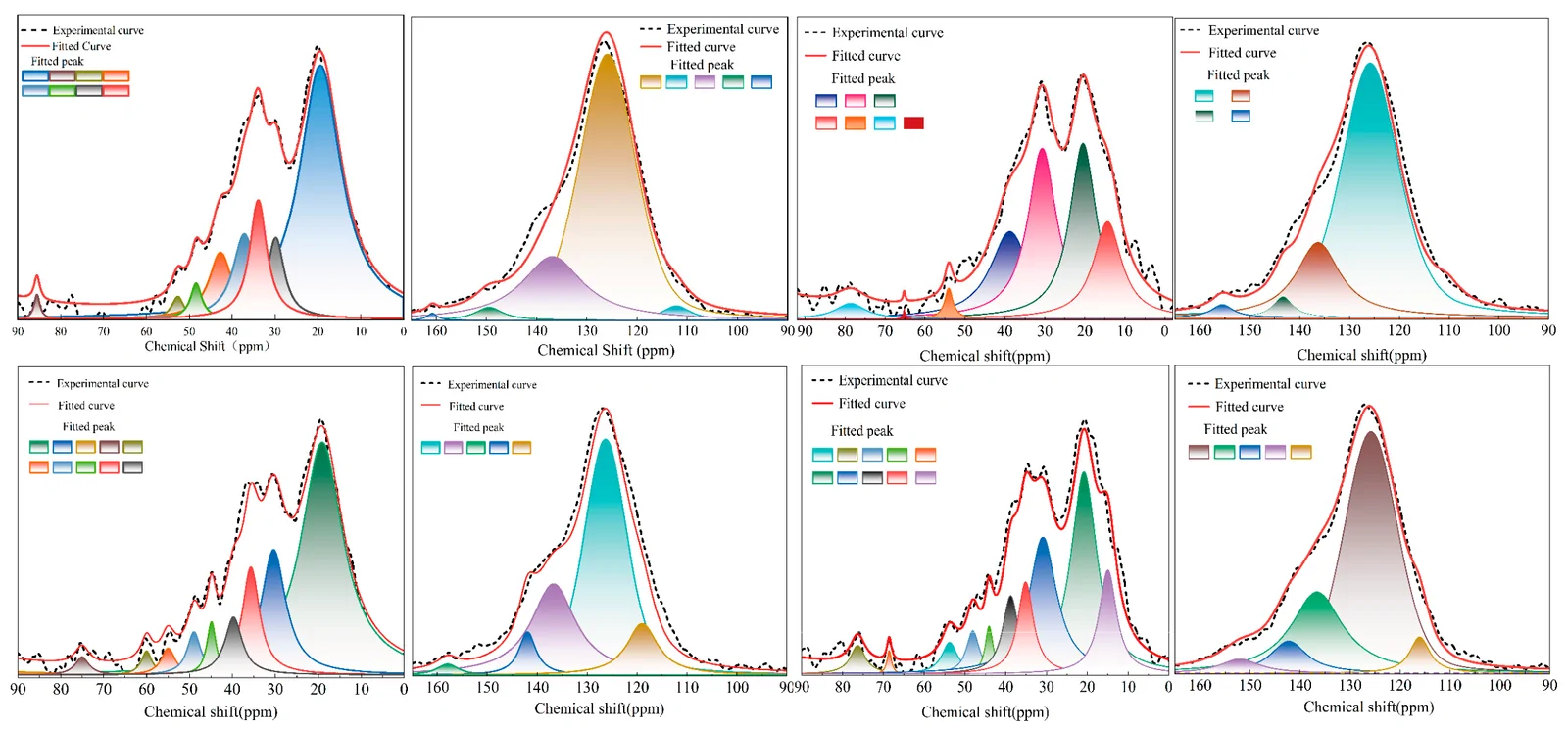
Curve-fitted ^13^C NMR spectra under sub-high temperature conditions (from left to right, the first row shows A0 and A1, and the second row shows A2 and A3).

Origin software was used to perform peak deconvolution of specific spectral regions in the FTIR and ^13^C NMR spectra of both sub-high temperature group samples and raw coal samples (Figure 7 and Figure 8). The resulting integrated peak areas were used to calculate the relative proportions of individual functional groups (Table 4). Moreover, the chemical shifts, peak assignments, relative abundances and structural parameters of functional groups in the coal matrix were obtained based on ^13^C NMR analysis (Table 5).

#### 3.2.1. Response Characteristics of Aliphatic Side Chains and Functional Groups Containing Oxygen

**Figure 9:**
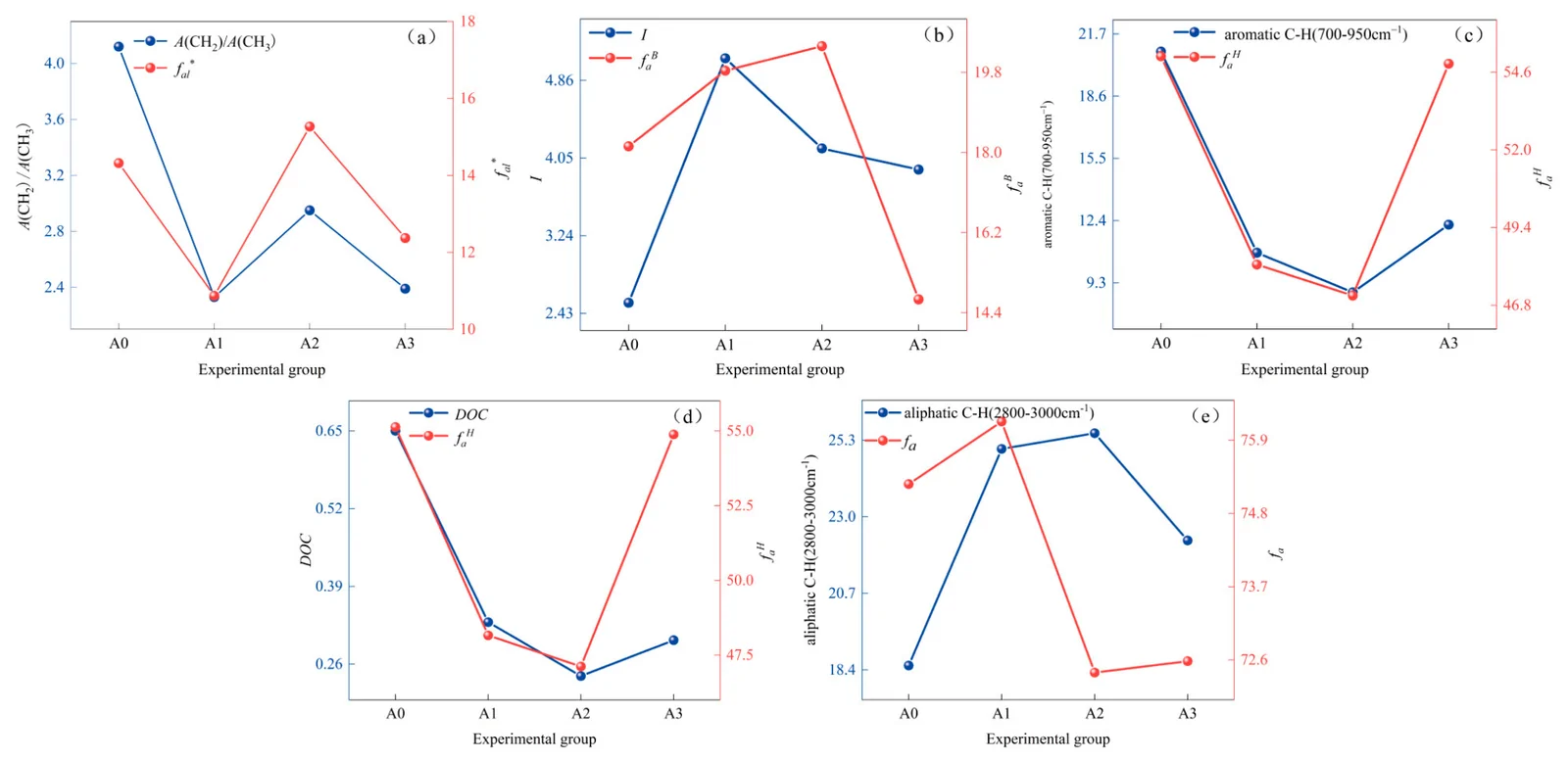
Correlation of shear rate with chemical structure variation at sub-high temperature. (**a**) the variation trends of parameters *A*(CH_2_)/*A*(CH_3_) and *f*_al_^*^; (**b**) the variation trends of parameters *I* and *f*_a_^B^; (**c**) the variation trends of parameters aromatic C-H (700-950cm^-1^) and *f*_a_^H^; (**d**) the variation trends of parameters *DOC* and *f*_a_^H^; (**e**) the variation trends of parameters aromatic C-H (2800-3000cm^-1^) and *f*_a_.

Among the FTIR parameters, the *A*(CH_2_)/*A*(CH_3_) ratio reflects the length and branching degree of aliphatic chains, with a lower value indicating shorter side chains or a higher degree of branching. As shown in Table 4, the original coal A0 had an *A*(CH_2_)/*A*(CH_3_) value of 4.12. After shearing was applied, this ratio dropped to its lowest point of 2.33 in A1, recovered to 2.95 in A2, and then decreased again to 2.39 in A3 (Figure 9a). In the ^13^C NMR data, the parameter corresponding to *A*(CH_2_)/*A*(CH_3_) is the methyl and quaternary carbon content (f_al_^*^), which represents the proportion of aliphatic carbon. The calculated results (Table 5) showed that the f_al_^*^ content in A1 decreased from 14.32% in the original coal to 10.87%, consistent with the trend observed in FTIR. This pronounced reduction in terminal methyl groups and side chains aligns with the highest CH_4_ yield (1.0 mL) and CO yield (0.15 mL) detected by GC–MS, suggesting that gas generation is closely related to changes in the aliphatic side chains. The proportion of aliphatic —C—H (2800–3000 cm^−1^) in A0 was 18.53%, increasing to 25.04% in A1 and reaching a maximum of 25.51% in A2, before declining slightly to 22.29% in A3, which nonetheless remained above the level of the original coal (Figure 9d). Compared with A0, all sheared and deformed samples showed an increased proportion of aliphatic —C—H.

In terms of the change in oxygen with functional groups, the ^13^C NMR data showed that there were additional changes in the oxygen-bonded aliphatic carbon content (*f_al_^O^*). A0 had a *f_al_^O^* content of 0.55% which increased to 1.58% when sheared at low speed (A1), 1.36% when sheared at A2 and 1.19% when sheared at A3. This value exhibited a slowly decreasing trend as the rate of shearing was increased but it was generally higher than that of the original coal.

#### 3.2.2. Structural Evolution of the Aromatic Skeleton

The aromatic —C==C— skeleton (1540–1650 cm^−1^) content of A0 was 32.13%, which progressively increased with rising shearing rate, reaching 32.87% in A1, 37.18% in A2, and 40.84% in A3. The content of the aromatic —C==C— skeleton thus exhibited a monotonically increasing trend with increasing shearing rate. In the ^13^C NMR data, the aromatic carbon content (*f_a_*), representing the percentage of aromatic structures in the carbon skeleton, was 75.24% for A0. As the shearing rate increased, *f_a_* rose to 76.18% in A1, then declined slightly in A2 and A3 to 72.41% and 72.58%, respectively (Figure 9b).

Regarding the FTIR data, the aromaticity index *I*, which describes the relative richness of the aromatic structures, increased to 5.09 (the largest value) compared to 2.54 in the raw coal (A0). The same tendency is proved by the ^13^C NMR results of bridgehead aromatic carbon (*f_a_^B^*)—a parameter showing the degree of condensation of the aromatic nucleus—which rose to 18.14% in A0 and then to 19.84% in sample A2. In the meantime, the percentage of alkyl-substituted aromatic carbon (*f_a_^S^*), which indicates the extent of side-chain attachment to aromatic rings, increased up to 5.50% rather than 1.64%. Conversely, another indicator of aromatic condensation, the protonated aromatic carbon fraction (*f_a_^H^*), reduced between 55.13 and 48.16; the size and direction of this reduction can be assumed as a measure of the degree of ordering or disruption of the coal macromolecular skeleton during rotational shearing. This structural change was noticed in the FTIR spectra as a decrease in the relative area of the aromatic —C—H out-of-plane bending band (700–950 cm^−1^) of 20.82% in the original coal and 10.80% in sample A1.

When the shearing rate reached a high speed (A3), the aromaticity *I* in FTIR dropped back to 3.93. Although still higher than that of the original coal, it showed a clear decrease compared with A1. In the ^13^C NMR data, *f_a_^B^*, representing the core of fused ring structures, dropped sharply to 14.70% in A3, well below the 18.14% of A0. At the same time, *f_a_^S^* fell back to 1.79%, approaching the A0 value, while *f_a_^H^* rebounded to 54.88%. The proportion of aromatic —C—H in FTIR also increased correspondingly to 12.20%.

*DOC* represents the degree of condensation of aromatic rings. The *DOC* of A0 was 0.65, which decreased to 0.33 and 0.24 at the A1 and A2 stages, respectively, corroborating the increase in *f_a_^H^* from the ^13^C NMR data (18.14%–20.39%). When the shearing rate reached a high speed (A3), the *DOC* value rebounded from 0.24 to 0.30, while the Har/Hal ratio, representing the ratio of aromatic hydrogen to aliphatic hydrogen, reached its maximum of 1.83 (Figure 9c), reflecting the exhaustion of aliphatic side chains.

### 3.3. Evolution of Chemical Structure Under Room Temperature Conditions

**Figure 10:**
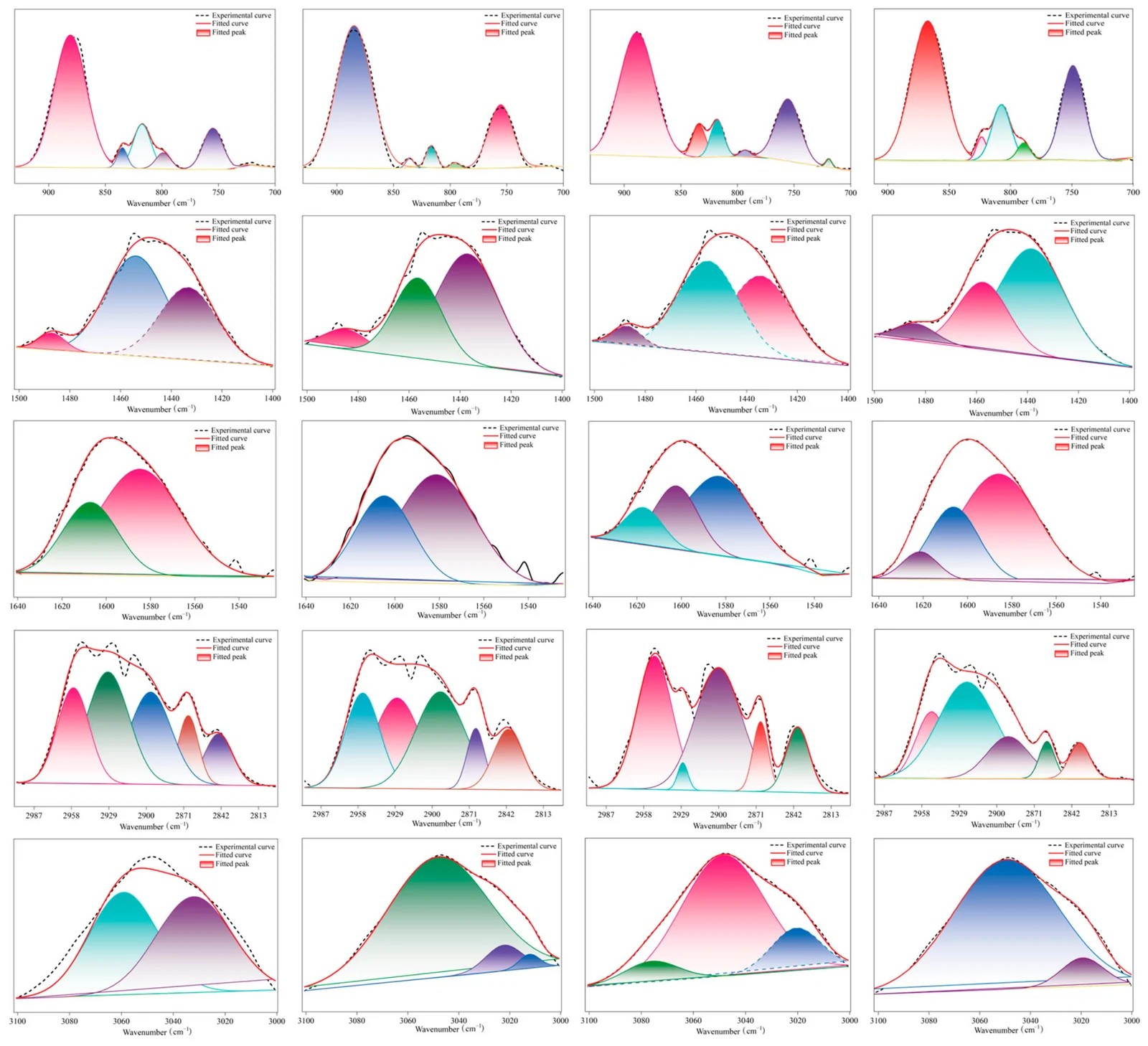
Curve-fitted FTIR spectra under room temperature conditions (from left to right, columns 1 to 4 are A4, A5, A6, A0 in sequence).

**Figure 11:**
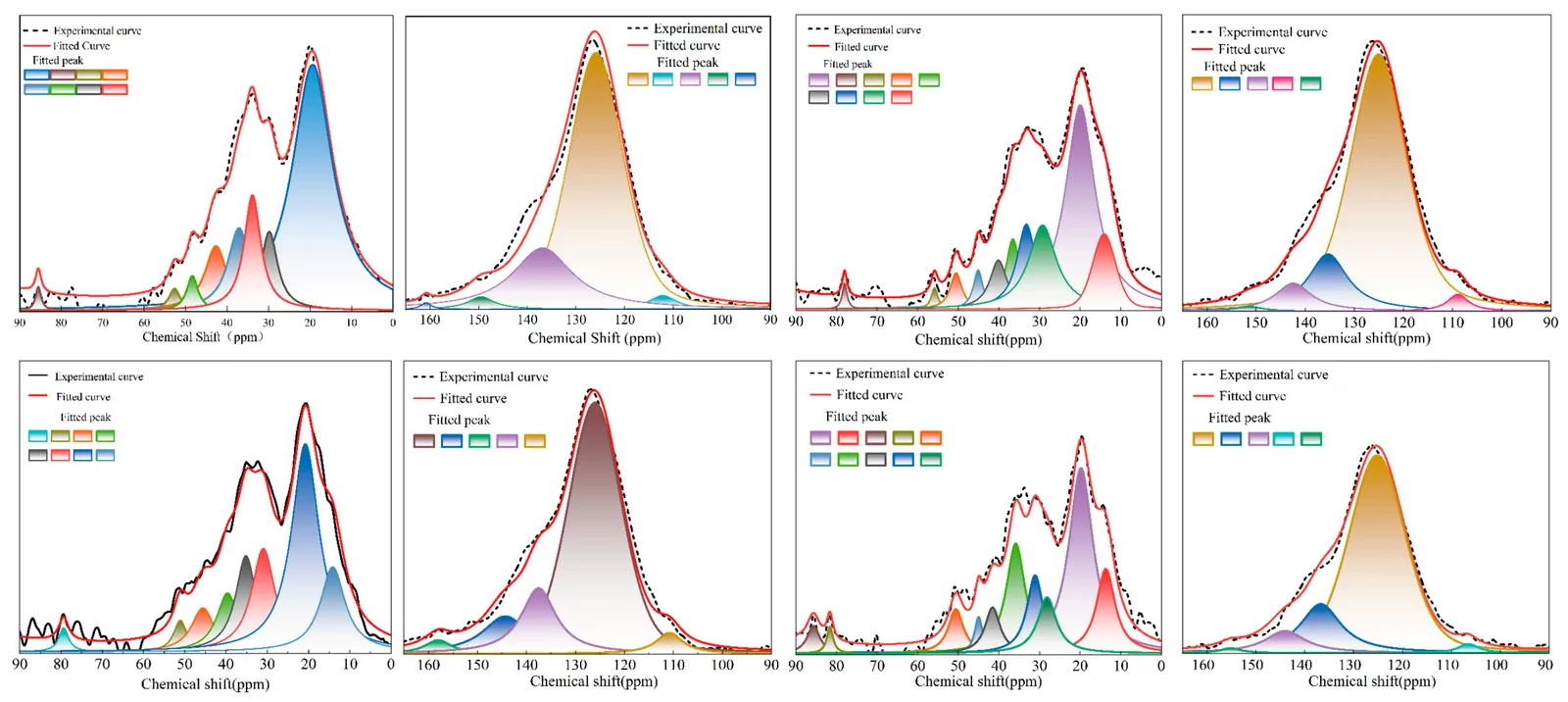
Curve-fitted ^13^C NMR spectra under room temperature conditions (from left to right, the first row shows A0 and A4, and the second row shows A5 and A6).

Peak fitting was performed on the FTIR and ^13^C NMR spectra of the room temperature group (Figure 10 and Figure 11), and the proportion of each functional group was calculated based on the peak areas obtained (Table 6). The peak positions, assignments, relative contents, and structural parameters of each functional group in the ^13^C NMR coal structure were determined (Table 7).

#### 3.3.1. Response Characteristics of Aliphatic Side Chains and Functional Groups

**Figure 12:**
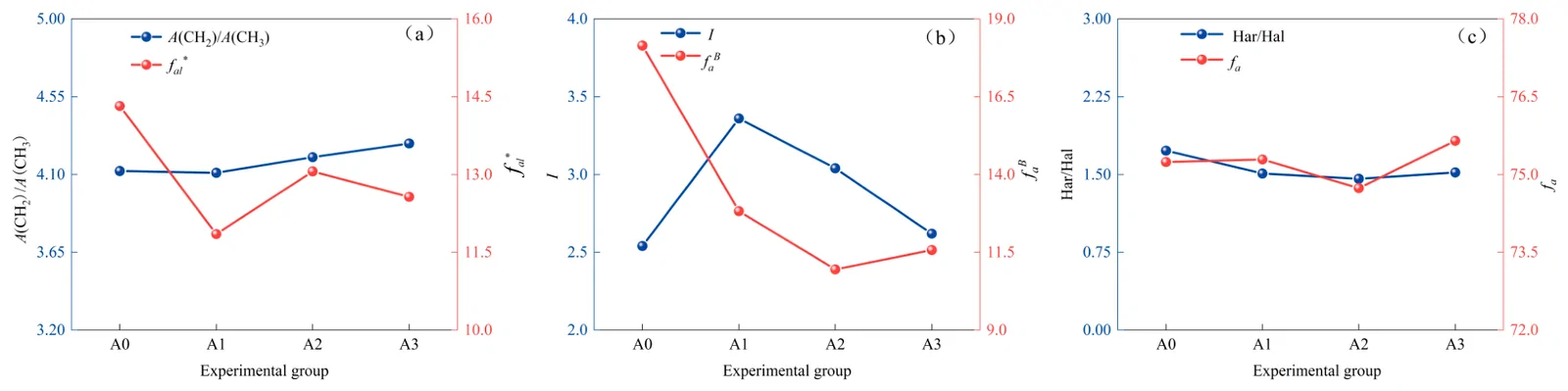
Correlation of shear rate with chemical structure variation at room temperature: (**a**) the variation trends of parameters *A*(CH_2_)/*A*(CH_3_) and *f*_al_^*^; (**b**) the variation trends of parameters *I* and *f*_a_^B^; (**c**) the variation trends of parameters Har/Hal and *f*_a_.

The *A*(CH_2_)/*A*(CH_3_) ratio for the original coal (A0) was 4.12. Following shearing at low, medium, and high rates (A4, A5, and A6, respectively), the corresponding values were 4.11, 4.20, and 4.28, exhibiting only marginal deviations from that of A0 (Figure 12b). In the FTIR spectra, the aliphatic —C—H stretching band (2800–3000 cm^−1^) of the raw coal contributed 18.53% to the total absorption. This fraction underwent a slight increase in samples A4 and A5, reaching 20.72% and 22.69%, respectively, thereby displaying an initial ascending trend. Nevertheless, when the shearing rate was further elevated to the highest level (A6), the proportion fell back to 19.58%, once more approaching the value recorded for the undeformed coal. With regard to the ^13^C NMR results, the *f_al_* values for all sheared specimens fell within a narrow interval of 24.35%–25.26%, showing negligible departure from the 24.76% measured for A0. The f_al_^*^ value of A0 was 14.32%, whereas those of A4, A5, and A6 were 11.85%, 13.06%, and 12.57%, respectively, pointing to a modest reduction induced by shearing. Conversely, *f_al_^H^* exhibited an upward shift, rising from 9.90% in A0 to 10.54% (A4), 10.98% (A5), and 10.73% (A6), with comparatively limited variation. Among all parameters examined, *f_al_^O^* underwent the most pronounced alteration under room-temperature conditions, climbing from 0.55% in A0 to 2.31% in A4 before decreasing progressively with further increases in shearing rate (1.22% in A5 and 1.04% in A6), although it remained elevated above the level of the original coal throughout.

#### 3.3.2. Structural Evolution of the Aromatic Structure

The aromatic carbon fraction (*f_a_*) of the original coal (A0) was 75.24, and after shearing at room temperature, the values were 75.29 (A4), 74.74 (A5), and 75.65 (A6), with only insignificant changes (Figure 12a). A0 had a bridgehead aromatic carbon content (*f_a_^B^*) of 18.14, which experienced a significant decrease upon shearing to 12.82 (A4), 10.95 (A5) and 11.57 (A6). Concurrently, the alkyl-substituted aromatic carbon fraction (*f_a_^S^*) rose significantly, increasing to 5.78% (A4), 4.89% (A5) and 8.65% (A6) compared to 1.64% in A0. The protonated aromatic carbon fraction (*f_a_^H^*) showed a non-monotonic trend where it first increased and then decreased: the values were 56.04% (A4) and 58.28% (A5), which were higher than that of A0 (55.13), and then decreased to 53.58% in the high-shear-rate group (A6).

The relative intensity of the aromatic —C==C— skeletal vibration band exhibited minor variations around the A0 value of 32.13% with the sheared samples giving 31.30% (A4), 33.21% (A5) and 29.84% (A6), but there was no obvious monotonic variation. In low-rate shearing (A4), the aromaticity index I, a measure of the degree of aromatic condensation, rose to 3.36 at A0 and then slowly decreased with shear rate to 3.04 (A5) and 2.62 (A6), but was still higher than the A0 baseline, thus showing an initial increase followed by a gradual decrease. This compositional shift is consistent with the concurrent shifts in substituent group distributions as indicated by the ^13^C NMR analysis. In comparison with the experimental group at 80 °C, the degree of aromatic condensation (*DOC*) values of the room-temperature group had irregular fluctuations (A4 = 0.56, A5 = 0.47, A6 = 0.75), but the overall level remained about that of the original coal (0.65).

In addition, this observation was further supported by the Har/Hal ratio, which is the ratio of aromatic hydrogen to aliphatic hydrogen. The original coal A0 had a Har/Hal value of 1.73, and at room temperature, with varying shearing rates, the values of A4, A5 and A6 were 1.51, 1.46 and 1.52, respectively. In contrast to the trend seen under the sub-high temperature condition of 80 °C, where the Har/Hal varied systematically with increasing shearing rate, the Har/Hal values of the room temperature group decreased relative to the original coal and showed only small and irregular variations with increasing shearing rate, without any obvious directional evolutionary trend (Figure 12c).

## 4. Discussion

### 4.1. Gas Generation Mechanism of Bituminous Coal Under Shearing Action

**Figure 13:**
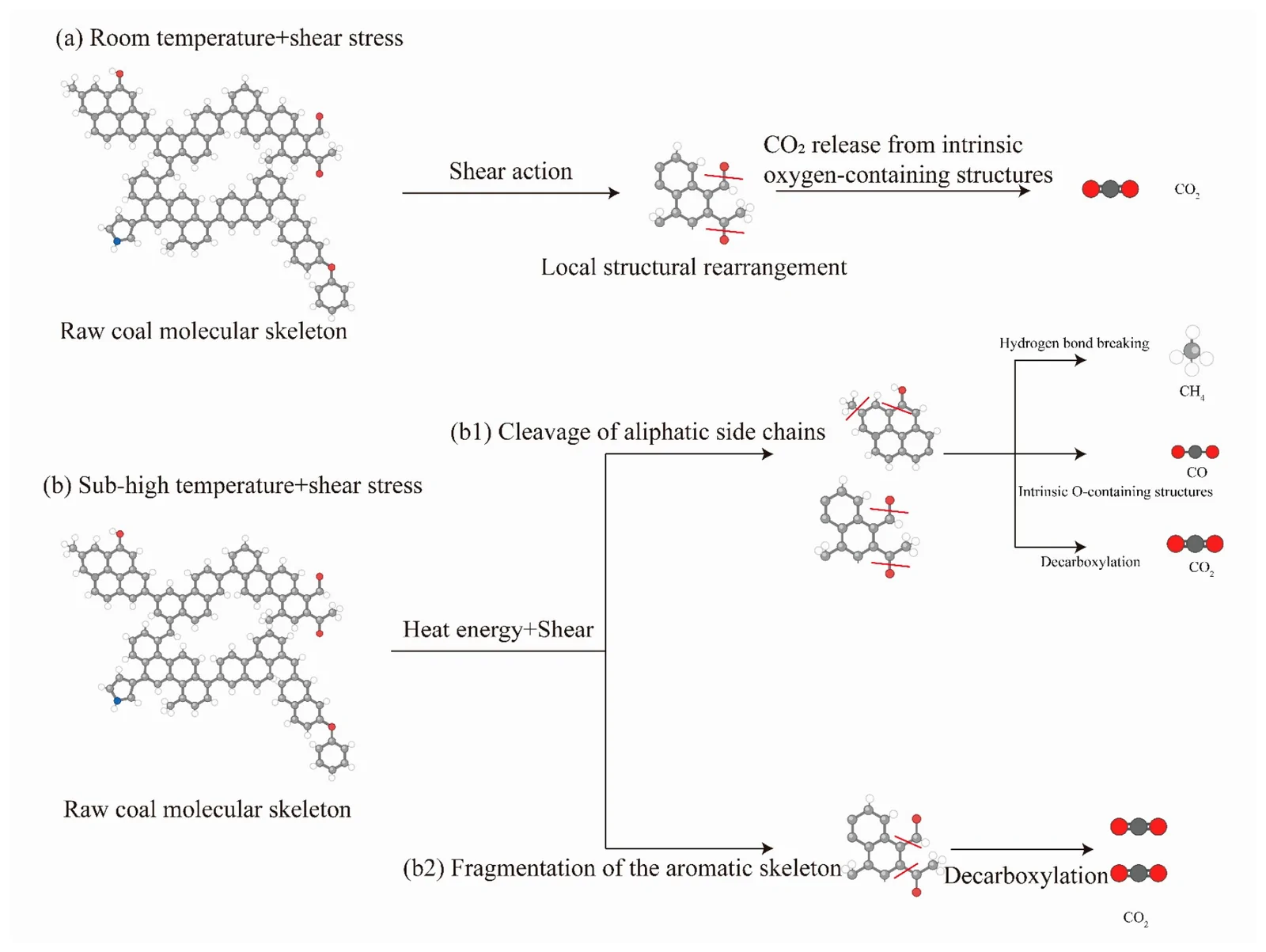
Schematic diagram of the gas generation mechanism during rotational shear deformation experiments.

Comparison of the rotational-shear experiments with the respective non-shear controls shows that shear has a great influence on gas release by medium-rank bituminous coal. At 25 °C, only CO_2_ was found and it increased with A4 to A6 (3.28 mL to 8.20 mL), but the non-shear control B2 released 1.64 mL CO_2_, *A*(CH_2_)/*A*(CH_3_) was near the A0 value, and the overall aromatic carbon fraction was about 75, which suggests that room-temperature shearing did not induce widespread cleavage of the bulk aliphatic or aromatic structure. However, *f_a_^B^* reduced significantly and *f_a_^S^* and *f_al_^O^* were significantly altered, indicating localized rearrangement of aromatic-edge and oxygen-containing structures. Since the experimental chamber was evacuated prior to shearing, the rise in CO_2_ cannot be explained by oxidation of external oxygen (Figure 13a) [26,29,42]. It is more plausibly connected with shear-facilitated release of existing CO_2_ and restricted breakdown or rearrangement of inherent oxygen-containing structures.

The emergence of CH_4_ and CO in the rotational shear groups, as opposed to the non-shear control dominated by CO_2_, at 80 °C suggests a more robust chemical response with thermal–mechanical coupling. In A1, CH_4_ had the highest value of 1.00 mL, *A*(CH_2_)/*A*(CH_3_) reduced to 2.33 and *f_al_* reduced to 10.87%. Past simulations have indicated that shear selectively influences weaker aliphatic side chains and bridge structures that are relatively weak [37,43,44]. Together with the relatively high abundance of aliphatic structures preserved in medium-rank bituminous coal, the current findings indicate that low-rate shear selectively activates terminal alkyl structures, producing methyl-containing radicals, which can be further stabilized as CH_4_ [35,45].

The A0 aromatic structure was also altered at A1. The overall aromatic carbon fraction, *f_a_^B^* and the FTIR aromaticity index were higher, and *DOC* reduced to 0.33–0.65. Thus, these alterations cannot be viewed merely as a condensation of stress-induced uniformity. They more aptly indicate relative enrichment and local rearrangement of aromatic domains after preferential transformation of peripheral aliphatic structures (Figure 13b1) [46,47,48].

There was a significant change between A1 and A2. CH_4_ reduced drastically between 1.00 and 0.16 mL and CO between 0.15 and 0.02 mL but CO_2_ increased between 3.00 and 6.74 mL. In the meantime, *A*(CH_2_)/*A*(CH_3_) and f_al_^*^ recovered, and *f_a_^B^* kept growing by 19.84 to 20.39%. Since FTIR and ^13^C NMR parameters are relative structural proportions, the recovery of aliphatic parameters does not imply the regeneration of previously cleaved side chains. Rather, A2 is probably a shift towards side-chain-controlled gas formation to greater structural redistribution and solid-phase rearrangement (Figure 13b2).

This transitional behavior also implies that various structural units in medium-rank bituminous coal do not respond simultaneously to the rising shear rate. The aliphatic structures are more sensitive at the first step of deformation, and the aromatic domains become progressively engaged with increasing mechanical input [49]. Thus, the non-monotonic changes in single FTIR and ^13^C NMR parameters cannot be considered as mere anomalous values but can indicate the heterogeneous and multistage reaction of the coal macromolecular network to rotational shearing.

The reaction pathway also altered at the high shear rate. In A3, CO_2_ was raised to 8.30 mL and CH_4_ and CO were reduced to trace levels. Simultaneously *f_a_^B^* declined drastically to 14.70, *f_a_^S^* declined, *f_a_^H^* recovered, *DOC* slightly increased to 0.30 and Har/Hal reached 1.83. These alterations reflect an increase in fragmentation of condensed aromatic structures and the creation of smaller aromatic units with more exposed edge sites [50,51]. Even though the aliphatic structural parameters also continued to change, CH_4_ almost vanished, indicating that bond cleavage is not the sole determinant of hydrocarbon yield.

To this end, the high CO_2_ yield at high shear rate can be mainly ascribed to the conversion of intrinsic oxygen-containing structures and not external oxidation. These structures can be cleaved and rearranged, possibly through decarboxylation, decarbonylation and internal oxygen redistribution to form CO_2_. The low level of CO can be attributed to the cleavage or rearrangement of inherent carbonyl-, ether- or other oxygen-containing groups under thermal–mechanical activation. Nevertheless, its gradual reduction between A1 and A3 indicates that there is no similar pathway in the formation of CO and CO_2_.

Furthermore, the speculations in this section are based solely on the indirect evidence from the relative variations in FTIR and ^13^C NMR parameters. No direct supporting evidence is currently available, and this is an important direction for our future research.

On the whole, the experiments at 80 °C show a progressive tectonic stress chemical reaction: low-rate shear promotes aliphatic-side-chain activation and CH_4_ formation, intermediate-rate shear promotes structural redistribution, and high-rate shear promotes aromatic-skeleton fragmentation and release of CO_2_ by intrinsic oxygen-containing structures. This progressive reaction is an indication of the unique mechanochemical response of medium-rank bituminous coal.

### 4.2. Implications for Coal and Gas Outbursts

Earlier research has indicated that the CH_4_ found in tectonically deformed coal is mostly attributed to physical processes, including compression of structural pores resulting in high gas pressure and migration of deep-seated gases, but whether tectonic forces can induce chemically based gas generation is an issue of continued debate [52,53]. However, tectonic stress is known to affect coal in two ways. First, mechanical energy transfer enhances shear-induced scission of aliphatic side chains, such as methyl and methylene groups, thus producing small-molecule free radicals or hydrocarbon gases [16,50,54]. Second, mechanical stress favors aromatic sheet alignment and polycondensation, which leads to aromatic enrichment and progressive ordering of the macromolecular structure [37,51,55].

The difference in rotational shear rates in this work is essentially a manifestation of the deformation mechanisms at different depths and strain regimes throughout tectonic development. Low-rate shearing (0.14 °/min) is associated with low-strain-rate ductile deformation under relatively deep geological conditions. At low strain rates, there is enough time to allow structural reorganization of coal macromolecules: stress is selectively transferred to cleave lower-energy aliphatic side chains and bridge bonds, removing methyl and methylene groups to form hydrocarbon gases such as the observed CH_4_. At the same time, the reduced strain rate allows aromatic sheets to accommodate stress through defect generation, which facilitates directional alignment and polycondensation of aromatic nuclei (stress-induced polycondensation) and increases overall structural ordering. On the other hand, high-rate shearing (3.44 °/min) models high strain-rate brittle deformation in the shallow crust. In this case, the rate of stress loading is much higher than the structural relaxation rate of coal macromolecules, resulting in rapid concentration of stresses on the aromatic skeleton. Without time to rearrange adaptively, the network of macromolecules experiences severe mechanochemical degradation, resulting in brittle rupture and ring-opening of the aromatic structure. These cracked skeletal fragments present very reactive free-radical sites that are easily oxidized under thermal–mechanical coupling, prevailing over significant CO_2_ generation and suppressing the production of alkane gas [56,57].

In these reactions, the structural changes in coal induced by tectonic stress involve covalent bond scissions, which require overcoming activation energy barriers that are hard to surmount at room temperature in order to initiate bond cleavage. In the experiments performed at room temperature in this study, either the thermal energy or shear stress was not sufficient to break the carbon atoms directly attached to the benzene rings. Rather, bond scission was mostly restricted to the aliphatic —C—H bonds of the alkyl side chains. Furthermore, the introduction of oxygen atoms onto aliphatic carbon atoms lowers the dissociation energy for bond scission, thereby promoting mid-chain scission of alkyl side chains and methylene bridges, followed by aromatization and polycondensation [58]. At ambient temperatures, irrespective of the shear rate applied, only CO_2_ was detected by GC–MS, with no definitive proof of extensive bond scissions (Table 3 and Table 7). This is typical of shallow crustal environments or brittle fault zones with low geothermal gradients. In such environments, the effect of tectonic stress on coal is predominantly physical; it is insufficient to surmount the activation energy barriers associated with chemical bond scissions and thus precludes the coal from undergoing the major structural changes necessary for gas generation.

Ambient temperature mechanical shearing does not supply enough energy to break C—C covalent bonds and, thus, physical fragmentation is the main process. Thermal input (80 °C), on the other hand, destabilizes the coal macromolecular structure, thus allowing the mechanical energy to be transformed into bond-cleavage energy and overcome the activation energy barriers [59]. The synergistic interaction between thermal energy and shear stress has been assumed to explain the high level of chemical transformations and gas generation in tectonically deformed coal at sub-high temperatures. This process gives an explanation of why field observations indicate that coal in tectonic zones often has metamorphic ranks and gas-bearing capacities that are higher than would be expected based on geothermal temperatures corresponding to their burial depths [60]. These results help to understand the mechanisms of gas generation that occur in medium-rank bituminous coal under the combined influence of rotational shear stress and temperature. Tectonic stress not only alters the physical characteristics of coal but also directly causes the chemical production of hydrocarbons. This study provides a vital theoretical basis of how tectonic stress actively contributes to the structural evolution of macromolecules and gas generation, which can provide practical insights into how deep coal and gas outbursts can be initiated and prevented, thus providing useful guidance on how safety can be improved in underground coal mining activities.

Even though the highest gas production of 8.31 mL (which is about 1 m^3^/t) under sub-high-temperature and high-shear-rate conditions confirms that tectonic stress directly causes gas evolution, there is still a difference of several orders of magnitude between this value and the adsorption capacity of medium-rank bituminous coal (typically 15–25 m^3^/t [61]) and the amount of gas released. Two main reasons explain this difference. First, significant variations in shear stress levels and total energy input are present. Deep tectonic zones experience very large in situ stress regimes, but the shear stress imposed in laboratory testing is only adequate to break a small fraction of weak bonds, such as aliphatic side chains and some aromatic bridge bonds, far below the energy level needed to cause widespread cleavage of condensed aromatic skeletons [62]. Second, time scale has a significant impact on the degree of gas generation. Tectonic evolution takes place on extremely long geological time scales, where protracted shear deformation provides coal macromolecules with enough time to undergo progressive structural reorganization and bond breaking. Conversely, laboratory studies cannot reproduce the effects of cumulative gas generation that occurs over geological timescales, thus explaining the discrepancy between experimental yields and the volumes of gas observed during outburst events.

## 5. Conclusions

Rotational shear deformation experiments conducted on medium-rank bituminous coal under varying thermal and mechanical conditions demonstrate the following:

(1) Thermal energy is the source of the activation threshold needed to cleave covalent bonds and form alkane. Shearing at 25 °C only causes physical comminution with CO_2_ being the only gas evolved. At 80 °C, thermal activation along with shear stress initiates the breaking of aliphatic side chains and oxygen functional groups leading to the formation of CH_4_ and CO.
(2) Low shear rates are the governing factors of side-chain detachment and polycondensation of aromatic nucleus. Shear stress selectively attacks low-energy aliphatic side chains, which is reflected by a significant drop in the *A*(CH_2_)/*A*(CH_3_) ratio, thus producing hydrocarbon gas (CH_4_). At the same time, this reaction enhances the directional alignment and polycondensation of aromatic nuclei, raising *f_a_^B^* to 19.84% in the raw coal and improving the overall structural ordering. Skeletal fragmentation and mechanical degradation, on the other hand, are controlled by high shear rates. Strong shear stress surpasses the strength limit of the aromatic network, causing skeletal cleavage. This structural collapse results in a rebound of the *DOC* value, lowers *f_a_^B^* to 14.70% and impels macromolecular disorder. In such circumstances, the pathways of hydrocarbon generation are inhibited, and the fractured skeletal fragments are instead thermo-mechanically oxidized, producing CO_2_ and CO.
(3) With the coupled parameters of sub-high temperature (80 °C) and low shear rate, the coal sample achieves the maximum level of structural ordering with the bridgehead aromatic carbon fraction (*f_a_^B^*) being at its maximum. This suggests that moderate thermal activation energy synergistically interacts with low strain rates to facilitate aromatic nucleus polycondensation, growing the aromatic layers and forcing them into closer proximity. This is supported by FTIR analysis: Group A1 has the highest aromaticity, showing that the detachment of aliphatic side chains effectively reduces steric effects. This side-chain cleavage does not affect the macromolecular skeleton but rather promotes the transformation of the coal structure to a more stable, highly ordered, graphite-like structure.
(4) This paper shows that the combination of sub-high temperature and low shear rate is favorable to the production and emission of alkane gases in medium-rank bituminous coal. Low-energy aliphatic side chains are preferentially attacked by shear stress, causing the detachment of methyl and methylene moieties and thus generating CH_4_. This removal of side chains facilitates the directional orientation and polycondensation of aromatic nuclei, which subsequently increases the overall structural ordering of the coal macromolecular structure. Conversely, with sub-high temperature and a high shear rate, the reaction pathway becomes dominated by oxidative decomposition of the aromatic skeleton, a regime where oxidative degradation and skeletal fragmentation are dominant processes, resulting in the formation of CO and CO_2_.
(5) Based on the indirect evidence obtained from FTIR and ^13^C NMR characterizations, this study deduced the reaction mechanism of medium-rank bituminous coal subjected to rotational shearing, established the correlation among shear intensity, macromolecular structural evolution and gas generation characteristics, and provided experimental support for understanding the hydrocarbon generation process in deep coal reservoirs and gas outbursts induced by tectonic stress.

## Figures and Tables

**Table 1:** Proximate and ultimate analyses of the coal sample.

Sample	Proximate Analysis/Mass%							Ultimate Analysis/Mass%					H/C
	*A*_d_	*V*_d_	*FC*_d_	*M*_ad_	*A*_ad_	*V*_daf_	*FC*_ad_	M	C(d)	H(d)	S(d)	O *(d)	
A0	10.7	20.6	68.5	1.52	10.6	23.17	67.5	0.41	78.9	4.27	0.35	16.07	0.05

*—obtained by difference method. ad—air-dried basis. d—dry basis. daf—dry ash-free basis. Ultimate analysis data are presented on a dry basis (d).

**Table 2:** Experimental conditions.

Group	Temperature/°C	Shear Rate/°/min	Axial Pressure/MPa	Time/h
A0	/	/	/	/
A1	80	0.14	12.5	48
A2		0.69		
A3		3.44		
A4	25	0.14		
A5		0.69		
A6		3.44		
B1	80	/		
B2	25	/		

**Table 3:** Statistics of gas generation during the experiments.

Group	Pressure Change/MPa	Actual Change/MPa	Total Gas Volume/mL	CO/mL	CH_4_/mL	CO_2_/mL
A1	0.05	0.03	4.15	0.15	1	3
A2	0.07	0.05	6.92	0.02	0.16	6.74
A3	0.08	0.06	8.31	0.004	0.0052	8.3
A4	0.03	0.02	3.28	/	/	3.28
A5	0.05	0.04	6.56	/	/	6.56
A6	0.06	0.05	8.2	/	/	8.2
B1	0.02	0.02	2.77	/	/	2.77
B2	0.01	0.01	1.64	/	/	1.64

Pressure change is the experimentally measured value. The remaining values for Group A have already had the corresponding adsorbed gas values deducted.

**Table 4:** Percentages and ratios of different FTIR absorption bands for the raw coal and sub-high temperature samples.

Sample	Aromatic—C—H (700–950 cm^−1^)	Aliphatic—C—H (1400–1500 cm^−1^)	Aromatic—C==C— (1540–1650 cm^−1^)	Aliphatic—C—H (2800–3000 cm^−1^)	Aromatic—C—H (3000–3100 cm^−1^)	*I*	*A*(CH_2_)/*A*(CH_3_)	*DOC*	Har/Hal
A0	20.82%	22.66%	32.13%	18.53%	5.86%	2.54	4.12	0.65	1.73
A1	10.80%	25.43%	32.87%	25.04%	1.55%	5.09	2.33	0.33	1.31
A2	8.83%	22.60%	37.18%	25.51%	5.44%	4.15	2.95	0.24	1.46
A3	12.20%	24.55%	40.84%	22.29%	4.88%	3.93	2.39	0.30	1.83

*A*(CH_2_)/*A*(CH_3_) = $\frac{A_{2920}}{A_{2950}}$. Aromaticity *I* = $\frac{A_{700–900}}{A_{2800–2900}}$. Polycondensation degree *DOC* = $\frac{A_{700–900}}{A_{1600}}$. Har/Hal = $\frac{A_{1540–1650}}{A_{2800–3000}}$.

**Table 5:** Percentages of different carbon types for the raw coal and sub-high temperature samples.

Group/Type	*f_al_*	f_al_^*^	*f_al_^H^*	*f_al_^O^*	*f_a_*	*f_a_^S^*	*f_a_^H^*	*f_a_^O^*	*f_a_^B^*	*f_a_^N^*
A0	24.76	14.32	9.90	0.55	75.24	1.64	55.13	0.33	18.14	20.11
A1	23.82	10.87	11.37	1.58	76.18	5.50	48.16	2.68	19.84	28.02
A2	27.59	15.27	10.96	1.36	72.41	3.71	47.12	1.18	20.39	25.28
A3	27.42	12.37	13.86	1.19	72.58	1.79	54.88	1.21	14.70	17.70

*f*_a_—aromatic carbon (90—165 ppm), including *f_al_^H^* (protonated aromatic carbon, 100–129 ppm) and *f_a_^N^* (non-protonated aromatic carbon, 129–165 ppm). *f_a_^N^* including *f_a_^O^* (oxygen-substituted aromatic carbon, 150–165 ppm), *f_a_^S^* (alkyl-substituted aromatic carbon (137–150 ppm), and *f_a_^B^* (bridgehead aromatic carbon, 129–137 ppm). *f_al_*—aliphatic carbon (0–90 ppm), including *f_al_^O^* (oxygen-bonded aliphatic carbon, 50–90 ppm), *f_al_^H^* (methylene and methane, 22–50 ppm) and f_al_^*^ (methyl carbon and quaternary carbon, 0–22 ppm).

**Table 6:** Percentages and ratios of different FTIR absorption bands for the raw coal and room temperature samples.

Sample	Aromatic—C—H (700–950 cm^−1^)	Aliphatic—C—H (1400–1500 cm^−1^)	Aromatic—C==C— (1540–1650 cm^−1^)	Aliphatic—C—H (2800–3000 cm^−1^)	Aromatic—C—H (3000–3100 cm^−1^)	*I*	*A*(CH_2_)/*A*(CH_3_)	*DOC*	Har/Hal
A0	20.82%	22.66%	32.13%	18.53%	5.86%	2.54	4.12	0.65	1.73
A4	17.39%	27.54%	31.30%	20.72%	3.04%	3.36	4.11	0.56	1.51
A5	15.57%	25.26%	33.21%	22.69%	6.39%	3.04	4.20	0.47	1.46
A6	22.52%	20.68%	29.84%	19.58%	4.26%	2.62	4.28	0.75	1.52

**Table 7:** Percentages of different carbon types for the raw coal and room temperature samples.

Group/Type	*f_al_*	f_al_^*^	*f_al_^H^*	*f_al_^O^*	*f_a_*	*f_a_^S^*	*f_a_^H^*	*f_a_^O^*	*f_a_^B^*	*f_a_^N^*
A0	24.76	14.32	9.90	0.55	75.24	1.64	55.13	0.33	18.14	20.11
A4	24.71	11.85	10.54	2.31	75.29	5.78	56.04	0.65	12.82	19.25
A5	25.26	13.06	10.98	1.22	74.74	4.89	58.28	0.62	10.95	16.46
A6	24.35	12.57	10.73	1.04	75.65	8.65	53.58	1.84	11.57	22.07

## Data Availability

The original contributions presented in this study are included in the article. Further inquiries can be directed to the corresponding author.
